# Occupational triangular fibrocartilage complex injury in a sewing machine operator

**DOI:** 10.2478/aiht-2025-76-4003

**Published:** 2025-09-30

**Authors:** Elif Reyhan Şahin, Mustafa Kahraman

**Affiliations:** Erzurum City Hospital Occupational Medicine Clinic, Erzurum, Turkey; Erzurum City Hospital, Radiology Department, Erzurum, Turkey

**Keywords:** arm-wrist score, grind test, job strain index, MRI, occupational injuries, repetitive wrist movement, grind test, indeks radnog opterećenja, MR, ocjena zgloba i podlaktice, ozljede na radu, ponavljajući pokreti zgloba

## Abstract

Triangular fibrocartilage complex (TFCC) injuries are associated with repetitive wrist movements and have mostly been reported in athletes but not in sewing machine operators, even though their jobs involve repetitive wrist movements. Our patient had operated a sewing machine for nine years across various workplaces. Two years ago, she began experiencing lateral ulnar pain, tenderness, and mild swelling in her left wrist. She was initially evaluated by her family physician, then by an orthopaedic surgeon, and was eventually referred to an occupational medicine specialist. TFCC compression test, TFCC stress test, grind test, and supination test were all positive. Ergonomic assessment showed an arm-wrist score of 5, a total rapid upper limb assessment (RULA) score of 5, and a job strain index (JSI) score of 13.5. Magnetic resonance imaging (MRI) of the left wrist showed changes consistent with a TFCC injury. Treatment included wrist immobilisation, oral painkillers, and rest, but her symptoms persisted upon return to the sewing machine, despite temporary use of a brace. Partial symptom improvement was observed only after job reassignment. This report presents the first confirmed case of a TFCC injury in a sewing machine operator and suggests that its potential occupational risks may have been overlooked in this population.

## Abstract (hr)

Ozljede triangularnog fibrokartilaginoznog kompleksa (engl. *triangular fibrocartilage complex*, krat. TFCC) povezuju se s ponavljajućim pokretima ručnog zgloba i uglavnom su opisane u sportaša, a u radnica na šivaćem stroju se ne spominju iako i njihovo zanimanje podrazumijeva ponavljajuće pokrete ručnog zgloba. Naša pacijentica radila je na šivaćem stroju devet godina na različitim radnim mjestima. Prije dvije godine počela je osjećati bol u ulnarnom dijelu lijevog ručnog zgloba, uz osjetljivost i blago oticanje. Najprije ju je pregledao liječnik obiteljske medicine, zatim ortoped, a na kraju je upućena specijalistu medicine rada. Testovi na kompresiju TFCC-a, test opterećenja TFCC-a, grind test i test supinacije bili su svi pozitivni. Ergonomskim pregledom utvrđena je ocjena 5 za oštećenje zgloba i podlaktice, ukupni RULA (engl. *Rapid Upper Limb Assessment*) iznosio je 5, a indeks radnog opterećenja (engl. *Job Strain Index*, JSI) iznosio je 13,5 bodova. Magnetska rezonancija (MR) lijevog ručnog zgloba pokazala je promjene u skladu s ozljedom TFCC-a. Obradom je pacijentici imobiliziran ručni zglob te su joj propisani oralni analgetici i mirovanje, no simptomi se nakon povratka na isto radno mjesto nisu povukli unatoč privremenom nošenju ortoze. Djelomično poboljšanje zamijećeno je tek nakon premještaja na drugo radno mjesto. Ovaj prikaz po prvi put opisuje potvrđeni slučaj ozljede TFCC-a u osobe koja radi na šivaćem stroju te upućuje na mogućnost previda potencijalnih profesionalnih rizika za ovu populaciju radnika.

Musculoskeletal disorders are disproportionately prevalent among women, potentially due to their dominance in occupations characterised by monotonous, repetitive, and physically demanding tasks, which are particularly common in operating a sewing machine (1, 2). Research indicates that sewing machinists suffer from musculoskeletal disorders due to poor working postures and repetitive hand and arm movements (3,4,5).

However, one musculoskeletal disorder that has hardly been reported in sewing machine operators is the triangular fibrocartilage complex (TFCC) injury. TFCC is a structure in the wrist that provides stability and support to the distal radioulnar joint (DRUJ) and the ulnocarpal joint. It consists of fibrocartilage, ligaments, and tendons that help cushion and stabilise the wrist during movements, particularly rotation and gripping. TFCC injury is most often caused by a trauma, repetitive stress, or degenerative changes resulting in wrist pain, especially on the ulnar side, clicking or grinding sensation in the wrist, weakness or instability with rotation, and swelling and tenderness over the wrist joint (6). It has been well-documented in athletes performing repetitive wrist-loading movements such as gymnasts, surfers, or wheelchair basketball players (7,8,9).

We therefore believe this is the first case report of a TFCC injury in a sewing machine operator in Turkey that may point to wider occupational health implications and be of interest to our colleagues, should they come across similar cases.

## CASE REPORT

A 45-year-old right-hand dominant female patient presented at our occupational medicine clinic with pain, tenderness, mild swelling, and warmth in her left wrist, which had begun about two years earlier. She denied any history of left-hand or wrist trauma. Her pain gradually worsened over time and became severe enough to disturb her sleep and interfere with performing daily tasks such as wringing clothes, opening jar lids, or shaking fabrics. Initially, she had been evaluated by her primary care physician, who recommended conservative treatment. She had used topical muscle relaxant creams, oral painkillers, and two to three courses of collagen supplements but had seen no significant improvement. She had no history or clinical evidence of any systemic disease, and was generally healthy, with no co-morbidities that could predispose her to TFCC pathology. She reported no previous trauma to her left hand or wrist and did not engage in sports or recreational physical activities involving repetitive wrist movement or weight-bearing stress which could have contributed to the TFCC injury.

Six months ago, she was examined by an orthopaedic specialist and was positive to the TFCC compression and supination tests. She was treated with a wrist immobilising splint for one week, followed by two weeks of rest before returning to work, where she resumed her sewing machine operations. At the time, she continued to use a wrist brace for another one to two months, but her symptoms persisted. Consequently, she was reassigned to a different position that did not involve sewing machine use, after which her symptoms partly improved. Based on her occupational history as a sewing machine operator and the positive physical examination findings, the orthopaedic specialist referred her to our occupational outpatient clinic with a suspicion of an occupational musculoskeletal disorder.

**Figure 1:**
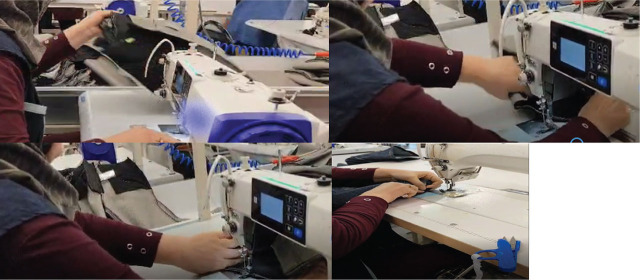
Repetitive hand and wrist movements performed by the patient during her daily sewing machine operation. These photographs illustrate various phases of the sewing process, including fabric positioning, guiding, and transferring

Her occupational history revealed a total of nine years operating a sewing machine professionally. At her last position at a denim manufacturing plant, her workday began at 07:30 and ended at 17:30, with two 15-minute breaks and a 30-minute lunch break. In an average workday, she handled 120–150 pairs of jeans per hour, delivered via a conveyor belt. For each item, she used her left hand to perform repetitive pronation and supination movements to properly position the jeans on the sewing machine for safety stitching. After stitching, she again rotated her left wrist to transfer the jeans to the next worker on the production line (Figure 1). This task was performed continuously, to handle 1,200–1,400 jeans a day. She worked five days a week without rotating shifts, and one to two hours of overtime one or two days a month.

**Figure 2:**
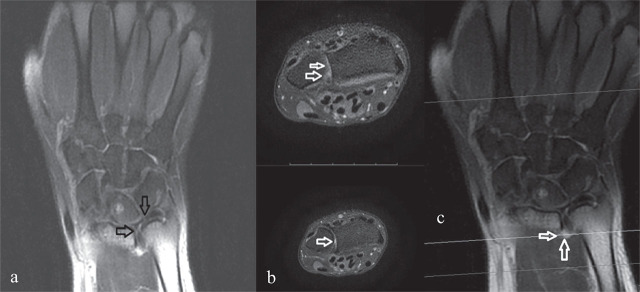
T2-weighted MRI of the left wrist showing: a) increased thickness and signal intensity of the triangular fibrocartilage complex (TFCC) (arrows); b) and c) joint effusion in the distal radioulnar joint (arrows) consistent with a TFCC injury

On physical examination at our clinic, the patient had ulnar foveal tenderness and pain with ulnar deviation and forearm rotation. In addition to the positive TFCC compression and supination tests performed earlier, TFCC stress and grind tests were also positive. Other systems were unremarkable. Magnetic resonance imaging (MRI) of the left wrist showed joint effusion in the distal radioulnar joint and the TFCC appeared thickened, with increased signal intensity consistent with the injury (Figure 2). An 8 mm lesion in the lunate bone showed hypointensity on T1-weighted images and hyperintensity on T2-weighted images, consistent with a bone cyst. The bony structures, muscles, and tendons of the wrist demonstrated homogeneous signal intensity. The joint spaces between the carpal bones were of normal width. No pathological findings were observed in the muscles or soft tissues within the imaging field.

Although the patient is right-hand dominant, her occupational posture mostly requires active use of the left hand during work. She did not report any symptoms in the right wrist, and physical examination of the right wrist revealed no abnormalities. Therefore, no comparative diagnostic assessment of the right wrist was deemed necessary. The patient reported that other workers performing similar tasks at the same workplace had also experienced comparable wrist symptoms and had received pharmacological treatment, suggesting a potential work-related pattern.

In subsequent ergonomic measurements, specifically the rapid upper limb assessment (RULA) and job strain index (JSI), the patient scored 5 and 13.5, respectively, confirming increased risk of musculoskeletal disorder development. The RULA scores of 5–6 point to the need for further examination and ergonomic modifications, while scores ≥7 indicate the need for immediate corrective action (10, 11). The JSI scores of 3–7 suggest moderate risk requiring further evaluation, and scores ≥7 indicate high risk (12). In our patient, the elevated RULA and JSI scores pointed to biomechanical stress imposed by repetitive tasks performed in a poorly optimised work environment.

Differential diagnosis ruled out alternative causes of the ulnar-sided wrist pain. Ulnar impaction syndrome was excluded based on neutral ulnar variance and the absence of subchondral bone changes on MRI (13). Lunotriquetral ligament injury and DRUJ instability were ruled out, because the ballottement and DRUJ stress and piano key tests were negative, and imaging confirmed preserved joint congruity and intact ligament structures (14). *Extensor carpi ulnaris* (ECU) tendinopathy, pisotriquetral arthritis, and hook of hamate fractures were excluded based on the absence of focal tenderness and normal imaging findings (15,16,17). Although the carpal tunnel syndrome can present with vague wrist discomfort, its hallmark features – paraesthesia in the thumb, index and middle fingers, nocturnal symptoms, and positive Tinel’s or Phalen’s signs (18) – were not observed in our patient. Following her job reassignment, the patient reported a marked improvement in her symptoms. Over time, her need for oral and topical analgesics decreased. She no longer experienced night-time wrist pain and reported that she was able to perform daily activities –previously limited by pain – without difficulty.

## DISCUSSION

This case, the first of its kind reported in Turkey, reveals a previously unrecognised occupational risk and calls for increased clinical attention. It underscores the need to consider TFCC injuries as a potential occupational risk in sewing machine operators, even though it has not been reported earlier in this population. Our clinical findings suggest that biomechanical demands inherent in such tasks may predispose these workers to TFCC pathology.

Accurate diagnosis requires careful occupational history-taking and targeted physical examination, particularly in high-risk occupational groups. Early identification is crucial, as TFCC injuries often present with non-specific symptoms that may be overlooked in routine assessments. This case highlights the importance of clinical vigilance and interdisciplinary collaboration in occupational health practice.

Furthermore, our case underscores the need for occupational health surveillance and proactive ergonomic strategies in industries with high repetitive load demands. The lack of ergonomic awareness and preventive training may exacerbate the risk of TFCC injuries in manual occupations such as sewing. Evidence suggests that ergonomic interventions – including improved wrist positioning, scheduled breaks, workstation redesign, and worker education – can significantly reduce the incidence of repetitive strain injuries (19, 20). In light of our patient’s ergonomic risk profile, we engaged with the workplace physician to recommend modifications aimed at reducing her biomechanical strain. Such proactive measures – encompassing ergonomic redesign, worker education, and task modification – are essential for preventing work-related musculoskeletal disorders and improving occupational well-being.

Finally, in accordance with national legislation, this case was formally reported as an occupational disease to the relevant authorities. Proper documentation and recognition of work-related health conditions not only support affected individuals’ rights but also contribute to broader preventive strategies and regulatory compliance.
